# Supplementary material for: Utilization of complementary and alternative medicine (CAM) by women with breast cancer or gynecological cancer
Source: PLoS One. 2023 May 12;18(5):e0285718. doi: 10.1371/journal.pone.0285718 (PMC10180689; doi:10.1371/journal.pone.0285718)
Supplement: Attachment — Submitted filename: Response to Reviewers 18Apr2023.docx [file pone.0285718.s001.docx]

# PLOS ONE: „Utilization of Complementary and Alternative Medicine (CAM) by Women with Breast Cancer or Gynecological Cancer”

Dear reviewers,

Thank you very much for the valuable comments and the constructive feedback. This is highly appreciated. In the following you will find the responses to all your remarks (indicated in red within the main manuscript).

Reviewer #1: I read with great interest the Manuscript titled "Utilization of Complementary and Alternative Medicine (CAM) by Women with Breast Cancer or Gynecological Cancer " which falls within the aim of the Journal. In my honest opinion, the topic is interesting enough to attract the readers’ attention. Nevertheless, authors should clarify some point and improve the discussion citing relevant and novel key articles about the topic.

- The whole text should be corrected by a native English speaker in order to make the work clearer and more readable.

-The introduction should be extended and completed. I find interesting a reference to:

D’Oria O., Golia D’Augè T., Baiocco E., Vincenzoni C., Mancini A, Bruno V., …A. Giannini The role of preoperative frailty assessment in patients affected by gynecological cancer: a narrative review Ital J Gynaecol Obstet. 2022 June p.p. 76-83 doi: 10.36129/jog.2022.34.

- Inclusion/exclusion criteria should be better clarified by extending their description.

- Discussions can be expanded and improved by citing relevant articles (I suggest authors to read and insert in references the following article PMID: 36141217).

Considered all these points, I think it could be of interest for the readers and, in my opinion, it deserves the priority to be published after minor revisions.

Many thanks for your feedback. The whole article has been reviewed regarding the language use.

The recommended review paper is very relevant with regard to the clinical practice. In addition, the use of CAM within our patient group is representing a strategy to improve the oncological outcome similar to the evaluation of the frailty of all gynecological tumor patients. The recommended publication has been included into the citation list.

The inclusion/exclusion criteria have been clarified by extending their description. Many thanks for the advice.

The recommended article about the progress regarding prevention and early detection of gynecological tumors was very helpful. We have extended the discussion with respect to recent relevant key publications.

Reviewer #2: The manuscript is perfectly fine, you may proceed. The manuscript needs no further correction on any ground, as per my opinion, in order to be published in the journal, unless as mentioned by other reviewers.

Thank you very much for the positive feedback.

Reviewer #3: The aim of this study is to investigate the use of CAM services in female patients with BC or GC. It is interesting to discuss the two types of cancer. However, the author did not tell the readers how the two are related. Why do they need to be discussed and compared together? What is the difference in socioeconomic status or background between the two? Even if it is understood that the quality of life of the two is related to the use of alternative medical care, does the author have any suggestions for medical providers to refer to? Overall, the structure of the entire article is loose, and the focus of discussion cannot be found.

Many thanks for your remarks. In Germany and many other countries breast cancer patients as well as patients with gynecological cancer are commonly treated by a specialist for gynecology. The medical care includes the tumor diagnostics, potential surgeries, execution of chemotherapeutic treatments, the full supervision of hormone therapies, completed by the cancer after-care. There is a strong common clinical ground for both types of cancer which is also reflected in many articles that report both tumor entities being investigated and compared as part of the same study. See also citations [17, 20, 29, 30, 32, 38].

We have included the following additional remark in the text: “patients with breast and gynecological tumors have a broad common clinical context with regard to diagnostics, therapy and after-care and are mostly treated by the same specialist. Therefore, both cancer types are discussed within the same framework.”

There are no differences between the two groups from a socioeconomic point of view, since all patients are health insured, which covers all general medical care costs but occasionally not all costs for the CAM. There are no differences with respect to the monthly income of both groups(see Tab.1)

The discussion has been extended by this point: “Adverse effects as well QoL scores respectivelyshould be investigated regularly before, during and after the cancer therapy preferably by using standardized questionnaires such as the EORTC QLQ-C30 or the QLQ-BR23. This approach would enable the evaluation of and recommendations for the use of CAM methods at a very individualized level.

Reviewer #4: I read with great interest the Manuscript titled “Utilization of Complementary and Alternative Medicine (CAM) by Women with Breast Cancer or Gynecological CancerUtilization of Complementary and Alternative Medicine (CAM) by Women with Breast Cancer or Gynecological Cancer", which falls within the aim of this Journal.

In my honest opinion, the topic is interesting enough to attract the readers’ attention. Methodology is accurate and conclusions are supported by the data analysis. Nevertheless, authors should clarify some point and improve the discussion citing relevant and novel key articles about the topic.

Authors should consider the following recommendations:

- Manuscript should be further revised by a native English speaker

- Inclusion criteria should be better clarified

- What are the actual clinical implications of this study? it is important to report the results obtained by the authors in the context of clinical practice and to adequately highlight what contribution this study adds to the literature already existing on the topic and to future study perspectives

- Does this manuscript conform the Enhancing the QUAlity and Transparency Of health Research (EQUATOR) network guidelines? It would be mandatory to declare about this element

- Was this study registered? I could not find any information about this point.

In light of the advanced techniques to detect early-stage disease, to date it is mandatory to consider even the possibility of fertility-sparing approaches in order to preserve reproductive potential of women affected by gynecological cancers. I invite authors to discuss this point, referring to: PMID: 22398708; PMID: 34769256.

- Accumulating evidence suggests that obesity and metabolic diseases may play a key role in increasing the risk of cancers, modulating pivotal cross-talk pathways for cell proliferation and differentiation. I recommend to stress these important points about prevention and screening strategies in gynecological oncology, referring to: PMID: 35314087; PMID: 36141217.

Thank you very much for the interesting feedback on our manuscript. The whole manuscript has been revised by a native speaker.

This study points to the importance of globally common definitions for CAM and CAM related methods with regard to the comparability of studies.

The focus on a large patient group within our study demonstrates to which extent CAM methods are used and which differences can be found between the two types of cancer patients. For doctors as well as for clinical staff, it is very important to know which preferences patients have regarding their aftercare within the framework of oncological therapy. It helps all physicians to define and recommend the right additional medical treatment for each patient individually, which can be easily integrated into clinical care plans.

In addition, the need for a prospective study design has been pointed out in order to be able to draw respective causal conclusions in the future.

In our view, the manuscript corresponds fully to the guidelines of the EQUATOR network. All data and results are provided in a fully transparent manner.

There has been no registration on a relevant platform, however the study protocol has been published as open access.

We have read the recommended studies with great interest. The possibilities of fertility friendly approaches are an important aspect in the context of the holistic care of female cancer patients, especially of young cancer patients. The focus of our work lies on the value and the benefit of CAM and shows specifically for the young patient group a significantly higher level of CAM use. Future studies could further investigate the correlation between CAM use and the fertility of young female patients. Unfortunately, no data have been revealed regarding this potentially interesting interdependency within this study.

We have also detected an interdependency between co – morbidities (i.e .adipositas, diabetes) and CAM usage. It became obvious that patients with co-morbidities made much less use (app. 50%) of CAM than patients with GC (data not shown). The potential of cancer prevention and screening methods needs to be communicated more accurately in the future. Additionally, more education/research becomes necessary about helpful but also harmful CAM methods within the primary prevention. Thank you for the helpful advice on literature.

Reviewer #5: Dear Authors,

This study is very interesting albeit limited, as it is a cross-sectional design and therefore, no conclusion can be drawn regarding causality with the use of CAM. including patients in different therapeutic contexts (intensive care clinic, outpatient clinics, rehabilitation centers), it is not possible to examine the use of CAM in a specific way, but the result has some bias due to the variables. While it must be recognized that since the methodology is identical, both types of cancer can be directly compared. I would have appreciated evaluations of predictive outcomes for the use of CAM, and its benefits for QoL.

Finally, for a multidisciplinary approach we propose to mention: DOI:10.3390/cancers14143457

Thank you very much for the constructive evaluation. In the cited articles it is also shown that patients and healthy risk groups respectively, as well as patients interested in CAM need and wish for more education about CAM provided by medical staff. In all cases, physicians as well as other medical staff need to be educated more intensively, a point that we have added to our discussion. We also agree with you on the importance of longitudinal studies. Only with those types of evaluations it will be possible to gain predictive results for CAM, including their influence on the QoL. This has been included in the discussion as well.
